# Community response to artemisinin-based combination therapy for childhood malaria: a case study from Dar es Salaam, Tanzania

**DOI:** 10.1186/1475-2875-9-61

**Published:** 2010-02-26

**Authors:** Vinay R Kamat, Daniel J Nyato

**Affiliations:** 1 Department of Anthropology, University of British Columbia, Vancouver, Canada; 2 Department of Political Science and Sociology, University of Dodoma, Dodoma, Tanzania

## Abstract

**Background:**

New malaria treatment guidelines in Tanzania have led to the large-scale deployment of artemether-lumefantrine (Coartem^®^), popularly known as ALu or *dawa mseto*. Very little is known about how people in malaria endemic areas interpret policy makers' decision to replace existing anti-malarials, such as sulphadoxine-pyrimethamine (SP) with "new" treatment regimens, such as ALu or other formulations of ACT. This study was conducted to examine community level understandings and interpretations of ALu's efficacy and side-effects. The paper specifically examines the perceived efficacy of ALu as articulated by the mothers of young children diagnosed with malaria and prescribed ALu.

**Methods:**

Participant observation, six focus group discussions in two large villages, followed by interviews with a random sample of 110 mothers of children less than five years of age, who were diagnosed with malaria and prescribed ALu. Additionally, observations were conducted in two village dispensaries involving interactions between mothers/caretakers and health care providers.

**Results:**

While more than two-thirds of the mothers had an overall negative disposition toward SP, 97.5% of them spoke favourably about ALu, emphasizing it's ability to help their children to rapidly recover from malaria, without undesirable side-effects. 62.5% of the mothers reported that they were spending less money dealing with malaria than previously when their child was treated with SP. 88% of the mothers had waited for 48 hours or more after the onset of fever before taking their child to the dispensary. Mothers' knowledge and reporting of ALu's dosage was, in many cases, inconsistent with the recommended dosage schedule for children.

**Conclusion:**

Deployment of ALu has significantly changed community level perceptions of anti-malarial treatment. However, mothers continue to delay seeking care before accessing ALu, limiting the impact of highly subsidized rollout of the drug. Implementation of ACT-based treatment guidelines must be complemented with educational campaigns to insure that mothers seek prompt help for their children within 24 hours of the onset of fever. Improved communication between health care providers and mothers of sick children can facilitate better adherence to ALu's recommended dosage. Community level interpretations of anti-malarials are multifaceted; integrating knowledge of local beliefs and practices surrounding consumption of anti-malarials into programmatic goals can help to significantly improve malaria control interventions.

## Background

In December 2006, Tanzania implemented new malaria treatment guidelines requiring the large scale deployment of Coartem^® ^(Novartis), a fixed-dose artemether/lumefantrine-based combination therapy (ACT) popularly known as ALu or *dawa mseto *in public health facilities, to treat uncomplicated malaria [1]. These new guidelines were implemented five years after the government decided to replace chloroquine (CQ) with sulphadoxine-pyrimethamine (SP) as the first-line treatment for uncomplicated malaria. Studies detailing how people in malaria endemic areas interpret policy makers' decisions to replace existing anti-malarials, such as SP, with "new" treatments, such as ALu or another artemisinin-based combination therapy (ACT), are lacking. Examining how adults and children use a newly introduced drug can inform the design of interventions aimed at improving drug use and therapeutic outcomes in community settings [2-4].

Documenting community level understandings and interpretations of ALu's efficacy and side effects is important for several reasons. First, the deployment of ACT on a large scale marks a major shift in global discourses on malaria control, mainly because ACT is a very expensive therapy that is unsustainable in poor countries like Tanzania without substantial donor funding [5,6]. Second, ACT is often described as "the key weapon" in the fight against malarial parasites because there are few affordable alternatives to ACT [7,8]. Third, researchers fear that malaria parasites could develop resistance to component drugs in ACT, due to the inappropriate use of artemisinin monotherapies [9,10]. Finally, medical anthropologists in particular have consistently pointed out that the perceived efficacy of a drug is embedded in culturally specific expectations. Thus, the perceived efficacy and side effects of anti-malarials must be examined in specific cultural or community contexts [11-13]. Although some researchers have examined community level perspectives on SP among populations severely affected by malaria [14,15], there is very little information on the community level perceptions of ALu after the Tanzanian government introduced it on a large scale. Understanding how cultural perceptions influence decisions regarding the use and consumption of anti-malarials, both "old" and "new," can provide valuable insights into how the delivery of newer treatment regimens can be better managed.

Determining the efficacy and side effects of anti-malarials is a complex task, both in biomedical and behavioural terms. This is especially true when the patients are young children who have been treated with anti-malarials, and mothers do the reporting on their children's behalf [16]. Many researchers have pointed out that there are significant discrepancies between reported consumption of anti-malarials, efficacy and detectable levels of the specific anti-malarial found in blood samples [3,14]. As such, the goal of this paper is to examine the perceived efficacy of ALu as articulated by the mothers of young children diagnosed with malaria and prescribed ALu. Though limited in its scope, this approach, which may be characterized as "interpretive," is important because the success or failure of an anti-malarial treatment policy will ultimately depend on the perceptions and understanding about the drug's efficacy at the community level [13]. Given that people's prior experiences and perceptions significantly influence the extent to which they "adhere" to the new ACT drug regimen, community level studies of perceptions of malaria and anti-malarials can provide useful perspectives on how people interpret the efficacy of "new" anti-malarials in light of their experience with "old" anti-malarials. In Tanzania, for example, researchers have documented that, while many people have a negative disposition toward SP, they are nostalgic when talking about CQ, emphasizing that they would be very pleased if they had access to CQ because it was an inexpensive drug, which brought immediate relief to the patient due to its antipyretic effect [14,16].

## Methods

### Study area and population

This study was conducted in the Chamazi administrative ward of Temeke District, Dar es Salaam (population: 3.5 million), Tanzania's commercial capital, which comprises of three independently governed municipalities - Temeke, Ilala and Kinondoni. Temeke district, with a population of 886,529 in 2007, and an area of 656 sq.km. is the largest of the three districts that comprise Dar es Salaam. Chamazi ward, which is located some 25 km south of Dar es Salaam's central business district, has two large villages -- Chamazi proper (pop. 10,000) and Mbande (pop. 8,000). A number of small villages and hamlets surround these two large villages. 85% of the local residents are Muslims. While the majority of the local residents identify themselves as Zaramo, there are substantial numbers of people in these villages who identify themselves as Makonde, Matumbi, Mpogoro, Ndengereko, Ngindo, Nyamwezi, Msukuma and Myao, among others. Cash income is scarce for many of the local residents whose economic base is subsistence-oriented farming. The completion of the all-weather road in 1996, which connects the trading town of Mbagala with Mbande village, marked the beginning of a new wave of migrants into this region, mostly from north-western and south-eastern Tanzania. The road facilitated the rapid transportation of people and goods between the villages and the city. The local health arena is pluralistic as villagers have access to municipal dispensaries - one of which is located in Chamazi village and the other in Mbande village. Both dispensaries are generally well staffed and well stocked. Additionally, in Chamazi ward, there are more than ten registered "traditional healers" (*waganga*), two licensed private practitioner's clinics, and 14 drug stores (*duka la dawa baridi*), which are managed by people without adequate formal training.

### Data collection

Data were gathered during four months of fieldwork (May to August of 2007) in the Chamazi ward. Additional follow-up research led by the second author was undertaken during the months of July and August 2009. Data were gathered using a combination of participant observation in the villages and at the health facilities, exploratory focus group discussions (FGDs) and semi-structured interviews with mothers of children below five years of age, who were diagnosed with malaria and treated with ALu less than two weeks prior to the interview. All interviews were conducted in Kiswahili with the help of an experienced female research assistant. The first author interacted with all the interviewees and was present during all the interviews. The second author conducted follow-up interviews, organized FGDs, made observations at the dispensaries, and translated the interviews from Kiswahili to English.

### Focus group discussions

FGDs were conducted in Mbande and Chamazi with six groups of six to eight mothers (total 42 participants). Two field assistants who were local residents initially approached mothers of young children who were diagnosed with malaria and treated with ALu at the local municipal dispensary, and invited them to participate in the FGDs. Those who were recruited at the dispensaries and were willing to participate in the FGDs were given further details about the study, the venue for the FGDs, and their role in generating important information for the study through discussions in small groups with the help of a moderator. Some of the key questions and topics addressed in the focus groups were: symptoms that prompt mothers to take their sick children to the dispensary; reasons why mothers delay seeking prompt treatment for their children when they have high fever; participants' opinion regarding the changes they have noticed in the quality of treatment for malaria since SP was replaced with ALu as the first-line drug at public health facilities; their perceptions regarding the side-effects associated with SP and ALu; their perceptions regarding the cost of dealing with childhood malaria; measures taken by members of the community to protect their children from contracting malaria; whether in their view, the malaria situation in their village has improved since the introduction of ALu, and what according to them needs to be done to minimize malaria's impact on their community.

### Semi-structured interviews

After reviewing the data from the FGDs and refining the semi-structured interview schedule, detailed interviews were conducted with 110 mothers whose children were treated with ALu for malaria during the past two weeks, in Mbande, Chamazi, and three adjoining villages -- Wembebamia, Kiponza and Kisewe. Mothers were randomly selected from a list that was prepared following initial contacts with them at the dispensaries. Only those who were willing to participate in the study were interviewed. Mothers were interviewed regarding the child who was under five years of age, diagnosed with malaria and prescribed ALu at the local municipal dispensary less than two weeks before the interview. Two children in the study sample had been treated with ALu followed by another anti-malarial, such as quinine (QN) or antibiotics.

Mothers were asked to describe the symptoms that had prompted them to take their child to the municipal dispensary; the time between the onset of symptoms and their decision to take the child to the dispensary; the advice they had received from the doctor or the nurse at the dispensary; the period they had waited before concluding that their child had recovered from his or her illness; whether their child had experienced any undesirable bodily side effects (*madhara*) after being treated with ALu, and if so, to describe the side effects. Mothers were also asked to describe their experience of treating their children with ALu, and how these compared with their experiences of SP. Finally, as a closing question, they were asked to express their thoughts on why they believed malaria persisted in their respective villages.

Additionally, interactions between mothers with sick children and the health workers at the municipal dispensaries, surrounding the dispensing of ALu, were observed, focusing mainly on the advice given by the nurse to the mothers at the dispensing counter.

### Data analysis

All mothers who agreed to participate in the study gave their oral consent for the interview. Interviews lasting about 30 minutes were recorded on a digital audio-recorder, transcribed verbatim in Kiswahili and key passages were later translated into English. Quantitative data from the recorded interviews were entered into a spreadsheet and processed using Microsoft Excel^®^. The authors reviewed all the interview transcripts and extracted segments and passages that called for a closer analysis, which were then manually encoded and analysed. "Text" or qualitative data from FGDs and interviews were first entered in MicrosoftWord^® ^and processed using ATLAS.ti 6.0 for key words and quotes, and themes. Notes from the field diary were incorporated into the analysis.

#### Ethical Review

Permission to conduct this study was given by the Tanzania Commission for Science and Technology (COSTECH Permit No. 2006-366-CC-2005-36). The Behavioural Research Ethical Board, University of British Columbia, and the Medical Research Coordinating Committee of the National Malaria Research Institute, Dar es Salaam gave ethics clearance for this study. Additional research and ethics clearance was obtained from the University of Dodoma.

## Results

Demographic information on the 110 mothers interviewed for this study is presented in Table 1. Of the children whose mothers were interviewed (index child), 51% were male and 49% were female. Their average age was 38 months (range three months to 60 months).

**Table 1:** Background information on mothers interviewed for the study (n = 110)

Residence	n	%
Chamazi Kwamkongo	30	27.27
Kiponza	13	11.83
Kisewe	29	26.36
Mbande Kijiji	31	28.18
Wembebamia	07	6.36
**Age**		
17-21	15	13.63
22-26	38	34.54
27-31	26	23.63
32-36	10	9.10
40-50	21	19.10
**Education**		
Nil	16	14.55
Primary school years 1-6	18	16.36
Primary school year 7	70	63.64
Primary school year 8	2	1.81
Secondary school Form 2	4	3.64
**Marital Status**		
Married	57	51.82
Unmarried	34	30.91
Widow	2	1.81
Divorced	17	15.46
**Religion**		
Muslim	91	82.73
Christian	19	17.27
**Number of Children**		
1	36	32.73
2	32	29.09
3	20	18.18
4	14	12.73
5-7	8	7.27

### Symptom recognition, waiting period and therapy-seeking

While early detection and access to prompt, affordable and effective treatment is regarded as the cornerstone of a successful malaria control strategy[17], in the present study, only 12% of the mothers had taken their child to the dispensary within 24 hours after noticing that he/she had a fever. 35% of the mothers had waited for two days (48 hours); 34% had waited for three days (72 hours) and the remaining 19% had waited between four and six days (92+ hours) before taking their child to the dispensary. While the majority (81%) of the mothers had treated their child's fever with a store-bought antipyretic such as Panadol^® ^(paracetamol) a small number (2.5%) had used Panadol^® ^in combination with SP. Another 2.5% had given their child SP albeit to no avail. 14% of the mothers had not given their child any medication before taking him/her to the dispensary.

Nearly 90% of the mothers mentioned high fever or persistent fever as the most important symptom influencing their decision to take their child to the dispensary. They denoted key symptoms by using terms and phrases such as *homa kali *(high fever) or *mwili ulichemka *(the body temperature was very high), (22.5%), *homa haishuki *(the fever wouldn't come down), (34%), *homa ilikuwa inatisha *(the fever was frightening) (6%), *hali iliharibika zaidi *(the condition worsened) (20%), *hali ya mtoto haikuboreka *(the child's condition did not improve), (7.5%), and *alichoka na kutapika *(he/she was exhausted and vomited) (2.5%). The remaining 7.5% had taken their child to the dispensary "without thinking too much about it," mainly to get the doctor's advice. Thus, the key symptom prompting mothers to consider taking their sick child to the dispensary is high fever that does not subside following treatment with an antipyretic. However, observational data gathered at the two municipal dispensaries revealed that in addition to persistent high fever, mothers also mentioned *alitapika *(vomiting), *aliharisha *(diarrhoea) and *ananyongea *(bodily weakness) as other symptoms that had prompted them to take their sick child to the dispensary.

### Treatment recall and perceived efficacy

While 80% of the mothers reported that their child was prescribed ALu along with an antipyretic, often recognized as Panadol^® ^or Panadol syrup (*Panadol ya maji*), 15% of them reported that their child was prescribed only ALu. The remaining 5% of the mothers said that their child was prescribed ALu along with Panadol^® ^and either a cough syrup or a packet of oral rehydration solution (ORS). Significantly, 92.5% of the mothers had taken their child to the dispensary only once. The remaining 7.5% of the mothers had gone to the dispensary at least twice because their child's condition had not improved even after completing the dosage. Four children in the study sample had received further treatment at the district hospital, and two others were taken to the Muhimbili National Hospital (MNH) located 30 km away.

Focusing on perceptions of ALu's curative efficacy, mothers were asked whether they believed ALu was an effective drug in the treatment of malaria. Responses to the above question, coded retrospectively as positive or negative revealed that 97.5% of the mothers had an overall positive disposition toward ALu. They confidently stated *dawa inafaa*, *amepona kabisa, anaendelea vizuri tu *(the drug is effective, the child has completely recovered, and is doing well) (55%); *anaendelea vizuri, anacheza na anachangamka vizuri *(the child is doing fine, and is very active) (42.5%) to indicate that their child had completely recovered following treatment with ALu. Two mothers were uncertain if their child had completely recovered. Significantly, the data in Table 2 reveal that although all the index children in the study were prescribed ALu at the local dispensary, in many cases mothers' knowledge and reporting of the dosage schedule was inconsistent with the recommended dosage for their children.

**Table 2:** Mothers' recall of ALu's dosage (n = 110)

Dosage recall	n	%
Twice a day for three days, six tablets in total	63	57.30
Three times a day for three days	11	10.00
Two times a day for five days	4	3.64
Two times a day for six days	3	2.72
Three times a day for six days	4	3.64
Two times a day for seven days	3	2.72
One tablet for seven days	5	4.55
Half a tablet, three times a day for seven days	3	2.72
Half a tablet, twice a day for seven days	2	1.81
Half a tablet, twice a day for two days	1	.90
Quarter tablet, three times a day for three days	2	1.81
One table a day for five days	2	1.81
Forgot the dose, cannot recall	7	6.36

Note: For children in the 4 months to 5 years age group, weighing between 5 and 14 kilos, the standard recommended dose of artemether-lumefantrine is one tablet, twice a day after a gap of 8 hours, for three days in total (Guidelines for the treatment of malaria, WHO 2006).

### Perceived efficacy and side effects of ALu compared with SP

94% of the mothers reported that they had not noticed any *madhara *or "undesirable" side effect in their child who was most recently treated with ALu. They emphatically stated that they had not seen any side effects and the drug had helped the child to recover completely. The remaining five mothers mentioned various side effects including the worsening of the child's fever. By contrast, when asked to compare their experiences with SP, a majority of the mothers recalled a range of undesirable *madhara *they believed were caused by SP such as *inachokesha*, *mtoto ana legea sana *(causes extreme exhaustion, the child become very weak), *homa haishuki, haipungui, iko pale pale wiki nzima *(the fever does not go away for a week), *inaleta vipele mdomoni, mapele mwilini *(it results in mouth ulcers and rashes on the body), *kuwashwa washwa *(there's itching all over the body) and *anakuwa na marengerenge *(he/she developes impetigo), *mtoto hatulii *(the child becomes restless). A 40-year-old mother of three children explained:

*My son became very weak after he was treated with SP. I thought to myself "Have we treated the illness or have we worsened it?!" It took about two weeks for him to return to his normal self. But last week when he had malaria, he was treated with ALu; he woke up in the morning and started playing as usual and his condition returned to normal*.

Perceptions of ALu's efficacy were closely tied to the perceived cost of dealing with a child's malaria episode. More than 90% of the mothers emphatically stated that ALu was far superior to SP because of its long-lasting effect, and also because the process of treatment-seeking was less expensive. A 27-year-old mother of a three-year old child contextualized her experience with ALu and SP as follows:

It's a lot better now because ALu really helps. Earlier you had to pay to get SP, which in any case did not help; the fever wouldn't go away so you had to take your child to the dispensary three or four times. By then you'll have exhausted all your money. But now it's different; the medicine is good. At the dispensary they also do a blood test. If your child is treated with ALu, he'll get better right away. The medicine of today is genuine (dawa za uhakika). If you use it once, you get better right away, so there's no need to go to the dispensary again and again.

Perceptions of the drug's efficacy were also reflected in statements about the expenses incurred in the treatment of childhood malaria. 62.5% of the mothers reported spending less money treating malaria than when SP was the first-line drug. Owing to the fact that ALu was prescribed to them free of cost at the local dispensary and treatment seeking did not involve multiple trips to the health facilities, they could "afford" to deal with a malaria episode. However, 30% said that dealing with malaria had become more expensive than before, although many of them added that while SP was a relatively inexpensive drug, they could not trust it because it took a lot longer for the drug to work and they had to make multiple trips to health facilities. The remaining 7.5% of the mothers said that they did not find any significant difference in the expenses incurred during the SP era and now.

### Advice

95% of the mothers had received at least some advice from the dispensary staff along with a prescription for ALu. 77.5% reported that they were told to continue giving Panadol^® ^to their child and to return to the dispensary if the fever did not go away after three days. Another 17.5% were specifically told to keep their house and surroundings clean by clearing the grass, and to use an ITN to minimize mosquito bites. The remaining 5% of the mothers stated that they had not received any advice from the dispensary staff. Observational data revealed that the dispensary staff, usually one of the nurses, spent an average of two minutes advising the mothers on how to use ALu and reminding them that they need to give their child plenty of water to drink during the treatment period.

### Perceptions of malaria's persistence

While all mothers who participated in the FGDs and nearly 90% of those who were interviewed said that they were satisfied with ALu's efficacy, they frequently exclaimed *malaria iko nyingi! *meaning that there was still a lot of malaria in their respective villages. Elaborating on their response, more than 75% of the mothers attributed the persistence of malaria in their respective villages to poverty and poor environmental conditions (*mazingira machafu*). A 40-year-old mother of four children explained:

We are poor so we don't have enough nets for everyone in the house. When our relatives come to visit us, we'll say, Ah! Alright, let the guests sleep there under the net, and I'll sleep on this side with my children without a net. Naturally the mosquitoes bite us and we get malaria. What can we do? We are poor. There are many large families in this village who have only one net, and many people sleep in places where there are no nets at all.

Mothers gave multiple responses to describe their efforts to minimize the impact of malaria on their lives -- use of ITNs to prevent mosquito bites (76.5%) and covering the bed with an ITN early during the evening; keeping surroundings clean (76%) and making sure that children wear a sweater in the evenings before going to bed to prevent mosquitoes from biting them (27.5%)

## Discussion

The Tanzanian government's decision to deploy ALu as the first-line anti-malarial on a large scale, mainly through public health facilities, is laudable from a public health point of view. This decision, however, also invites more attention to how communities that are affected by malaria interpret the efficacy and side effects of newer and older anti-malarials. Monitoring how the introduction of new anti-malarials affects people's treatment expectations, the cultural meanings they attribute to old and new drugs, their reckoning of the cost factor in their search for therapy, and their responses to uncertainty in the context of poverty is critical for the successful deployment of new anti-malarial regimens.

The data from this study suggest that even though mothers are aware that they have access to a highly effective anti-malarial free of cost, the majority (88%) of them do not rush their child to a health facility for diagnosis and treatment within 24 hours of the onset of fever. Instead, they first treat their febrile child with a store-bought antipyretic to see if the fever subsides. They continuously monitor and evaluate their child's fever for up to three days, and in some cases for up to six days, before deciding to take him/her to a health facility. The data also suggest that self-treatment of febrile children with a store-bought anti-malarial in the Dar es Salaam region is uncommon. This observation is consistent with findings of recent studies in the Tanzanian context, which have reported that unlike during the chloroquine era when self-medication was the norm, there is a noticeable reluctance among the people of Tanzania to use a store-bought anti-malarial to treat childhood malaria as a first resort [14,18-20].

The data also suggest that mothers who participated in FGDs and those who were interviewed for this study were satisfied with ALu's therapeutic efficacy as well as what it costs them to access the drug; they were equally pleased with the fact that the personnel at the local dispensary perform a blood test (*vipimo*) on their children to confirm that they have malaria before prescribing ALu. While this often leads to longer waiting periods, not one mother in the study complained about the extended waiting periods at the dispensary. Thus, improved perception of ALu is related to improved perception of the quality of care exemplified by blood tests for malaria. These data are striking when compared to mothers' responses regarding sick children to a similar question pertaining to treatment with SP in a previous study in the same research setting [16]. In the previous study, 32% of the mothers were not satisfied with the treatment that their child had received at the first place of medical consultation and nearly 50% of the mothers attributed their child's recovery from the illness to a medicine/treatment other than SP given at the local municipal dispensary.

For the majority of the mothers interviewed for this study, the blood test marks a significant departure from the SP era when children were routinely/clinically diagnosed with malaria and presumptively prescribed SP, even as the majority of the mothers deemed it a useless and/or a dangerous drug. In addition to efficacy, cost entered the evaluation of anti-malarials. During the SP era, dealing with childhood malaria was usually an expensive undertaking. In the previous study up to 59% of the mothers had consulted more than one health facility in search of an alternative therapy for their sick child. In the process they had incurred additional expenses and lost precious time [16]. The financial burden increased exponentially from CQ to SP for mothers of febrile children, especially those who lacked a strong support network to help them out during a health crisis. Faced with repeated treatment failure, mothers sought treatment from multiple sources, incurring additional costs and other burdens. By contrast, the introduction of ALu has significantly changed the situation, as most of the mothers who participated in this study believed that they were spending significantly less money on dealing with a malaria episode than before. Although mothers identified ALu as cost-effective, as noted earlier, the majority of them had delayed in bringing their sick children to a health facility because they thought they were dealing with an ordinary fever (*homa ya kawaida*) or teething fever (*mtoto anaota meno*). Most of the mothers had decided to "wait and see" (*unasubiri ukimtazamia*) if the fever would go away following treatment with a store-bought antipyretic. These observations are significant in the context of recent discussions and debates surrounding the accuracy of malaria diagnosis, misdiagnosis, over-diagnosis, and the question whether to treat all fever cases presumptively with an anti-malarial or to rely on laboratory-confirmed diagnosis and treatment [21,22]. On the one hand, it may be argued that mothers who resort to a store-bought antipyretic and engage in a "wait and see" approach before deciding whether to rush the child to the dispensary or not, may in fact be minimizing the chances of their child being wrongly diagnosed and unnecessarily prescribed an anti-malarial. On the other hand, it may be argued that the ease with which mothers are able to obtain ALu, a highly effective anti-malarial at the dispensary, free of cost, may in fact be a key deterrent in their decision to rush their febrile child to the dispensary within 24 hours of the onset of fever. Experienced mothers in Dar es Salaam know fully well that if their child's condition were to worsen, they would most certainly get ALu at the dispensary that would enable their child to recover rapidly.

While plans are being implemented to provide the public with better access to ACT, there is an urgent need to implement socio-cultural and behavioural interventions that would persuade mothers to bring their sick children to a health facility for diagnosis and treatment within 24 hours of the onset of symptoms, and not wait for three or more days to see if the fever would subside with an antipyretic. This would minimize childhood mortality resulting from other severe febrile illnesses, such as pneumonia or meningitis, which cannot be easily managed at home [23]. Concurrently, these behavioural interventions will have to be accompanied by "technical" interventions to ensure more accurate diagnosis and appropriate treatment of febrile children. Further, health care providers need to be better trained to communicate more effectively with mothers whose children have been diagnosed with malaria. Many studies have reported that health care providers, especially in public health facilities in Tanzania, do not communicate well with their patients, as they frequently fail to inform them of the nature of the illness and details of the prescription [24,25].

This is especially true in situations where, due to shortage of drugs, health care providers may give Coartem^® ^blister package meant for adults to mothers, asking them to break up the tablets into two or four parts, and give them to their febrile children. In the present case, the discrepancy between the recommended dosage and schedule for ALu and the mothers' reporting of the dosage and schedule they adhered to (see Table 2), may be due to a combination of poor adherence, reporting problems, problems in recall, and insufficient communication between the health staff at the dispensary and the mothers. However, it is important to address the issue of discrepancy between recommended dosage and schedule and the patient's adherence to the correct drug regimen because partially effective treatment may result in recrudescence of the infection, and in the long run, contribute to the development of anti-malarial drug resistance [3,26,27].

This study has some limitations that should be considered. First, the study was conducted in a region and among a population that is relatively well served by the health care system, and where people have access to ACT. Caution must be exercised in extrapolating the findings of the study to other regions of Tanzania where there are remarkable differences in population configurations, health infrastructure, and people's access to ACT. Second, the sample size is relatively small to make major statistical inferences. Third, a bulk of the data analysed for this study is derived from narrative interviews with mothers. It was beyond the scope of the study to verify their reports about the drug's efficacy through other measures, including ascertaining drug levels in blood samples. The findings of this study are, therefore, context-based and limited in their generalizability. However, the findings of this study provide valuable insights into how community level interpretations of newly introduced anti-malarials can inform further research and policy decisions aimed at improving the coverage and delivery of ACT among economically vulnerable populations.

## Conclusion

The deployment of ALu in public health facilities for treatment of uncomplicated malaria has significantly altered people's perceptions of anti-malarial treatment. In the present study, the majority of the mothers not only regarded ALu as an effective anti-malarial, they also found that it significantly reduced their expenditure on dealing with a malarial episode because it did not require them to go through multiple treatment stages as was common during the SP era. However, the majority of the mothers delayed before accessing ALu, limiting the impact of the subsidized roll out of these drugs on health outcomes. Current efforts to make highly subsidized ACT more readily available through private retail pharmacies may result in patients being promptly treated with a highly effective anti-malarial. However, this complex intervention may also result in people's overdependence on the commercial sector for treatment of childhood fevers, and lead to additional financial burden on poor households [25,28]. Further, the results highlight the importance of educational campaigns to refine prompt treatment-seeking messages targeted at families by taking into account community beliefs and practices. It is important to continuously monitor people's discourse on treatment decisions, alternative courses of possible action, and to document how they interpret the efficacy and side effects of anti-malarials that are deployed as first-line drugs, and their treatment expectations and perceptions of medicine compatibility. At a time when approaches to dealing with malaria are becoming increasingly treatment-oriented, community-based behavioural research can remind us that the efficacy of anti-malarials is multifaceted. It is one thing to demonstrate the *in vivo *clinical or pharmacological efficacy of various anti-malarials in controlled environments, and quite another to ensure the effectiveness of the drugs in "real-life" situations [29]. In other words, anti-malarials, which reveal excellent efficacy under controlled clinical trial conditions, may not demonstrate equally excellent "effectiveness" when they are deployed widely under real-life conditions [3]. Successful delivery of effective malaria treatment requires that health planners do not downplay the broader socio-cultural, economic, technical, and political environments in which treatment regimens are implemented [30]. Thus, a lot more is at stake in malaria control than the rolling out of highly subsidized, highly efficacious ACT. Integrating knowledge of local beliefs and practices surrounding consumption of anti-malarials into programmatic goals can be immensely valuable in improving the rigor and effectiveness of malaria control interventions [31].

## Competing interests

The authors declare that they have no competing interests.

## Authors' contributions

DJN supervised data collection, translated the interviews, contributed to data analysis and write-up of the manuscript. VRK conceived of the study, participated in its design and coordination, carried out fieldwork, and drafted the manuscript. All authors read and approved the final manuscript.
